# Supplementary material for: Cell-type targeted CRISPR/Cas9 Clock knockdown in mouse VTA dopamine neurons alter sleep, behavior, and cellular excitability
Source: bioRxiv. 2026 Jun 25:2026.06.03.730017. Preprint. [Version 2] doi: 10.64898/2026.06.03.730017 (PMC13321094; doi:10.64898/2026.06.03.730017)
Supplement: Supplement 1 [file NIHPP2026.06.03.730017v2-supplement-1.pdf]

bioRxiv preprint doi: https://doi.org/10.64898/2026.06.03.730017; this version posted June 25, 2026. The copyright holder for this preprint (which was not certified by peer review) is the author/funder, who has granted bioRxiv a license to display the preprint in perpetuity. It is made available under a CC-BY 4.0 International license.

Supplemental Figures

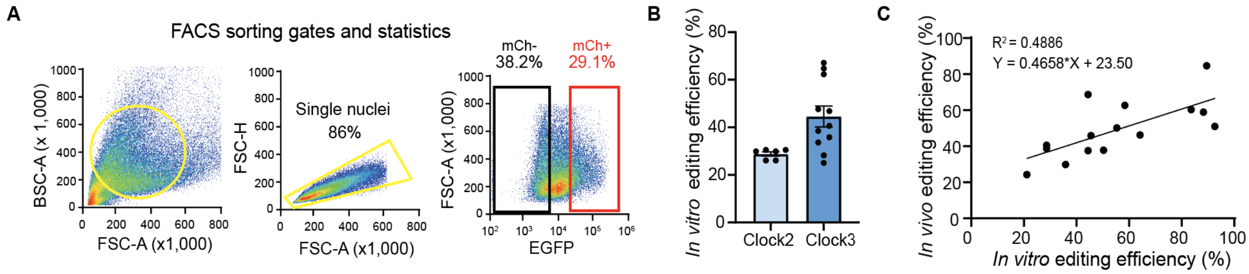

Supplementary Figure 1. (**A**) Representative FACS gating strategy and sorting statistics for NIH3T3 cells. (**B**) *In vitro* editing efficiency for Clock sgRNA #2 and #3. Mean editing efficiencies were 28.8% for sgRNA #2 and 44.6% for sgRNA #3. (**C**) Correlation between *in vivo* and *in vitro* editing efficiencies across sgRNA candidates. Each dot represents one sgRNA candidate. Linear regression and coefficient of determination are shown (R^2 = 0.4886, P = 0.0037, y=0.4658x+23.50).

bioRxiv preprint doi: https://doi.org/10.64898/2026.06.03.730017; this version posted June 25, 2026. The copyright holder for this preprint (which was not certified by peer review) is the author/funder, who has granted bioRxiv a license to display the preprint in perpetuity. It is made available under a CC-BY 4.0 International license.

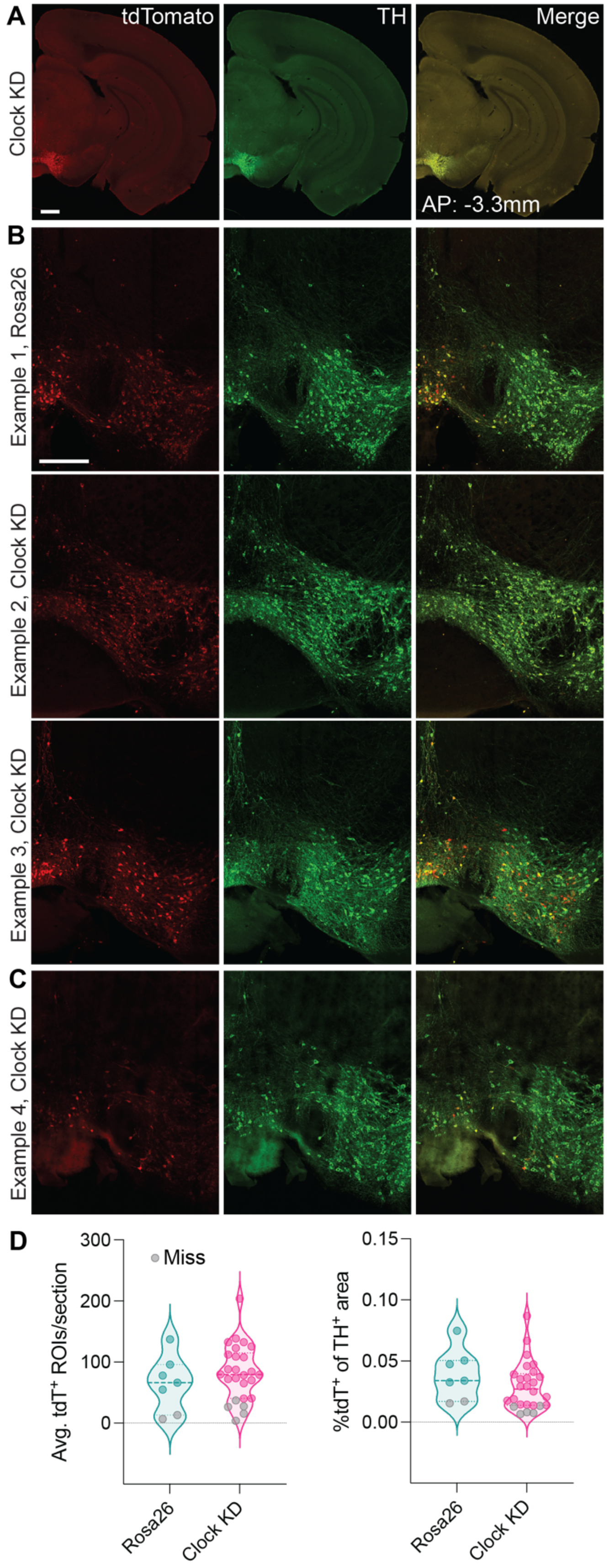

Supplementary Figure 2. (**A**) tdTomato and tyrosine hydroxylase (TH) expression in a neonatally transduced Clock KD mouse. Scale bar, 500 µm. (**B**) Same as **A**, but example 1 is of a control animal and examples 2-3 are Clock KD mice. Scale bar, 250 µm. (**C**) tdTomato and TH expression of a Clock KD animal considered a missed injection and therefore not included in the behavior dataset. (**D**) Quantification of the number of tdT+ regions of interest (ROIs) per 60 µm section of the VTA (left). 2-3 sections were imaged per mouse and the number of tdT+ ROIs was averaged per section. Quantification of the percent of TH+ area that is also tdT+ for a single VTA section (right). Grey circles, using the threshold of the bottom quartile in the quantification on the left, are considered missed injections and not included in the behavior data set. Rosa26: n = 7; Clock KD: n = 26. No statistically significant difference between groups.

bioRxiv preprint doi: https://doi.org/10.64898/2026.06.03.730017; this version posted June 25, 2026. The copyright holder for this preprint (which was not certified by peer review) is the author/funder, who has granted bioRxiv a license to display the preprint in perpetuity. It is made available under a CC-BY 4.0 International license.

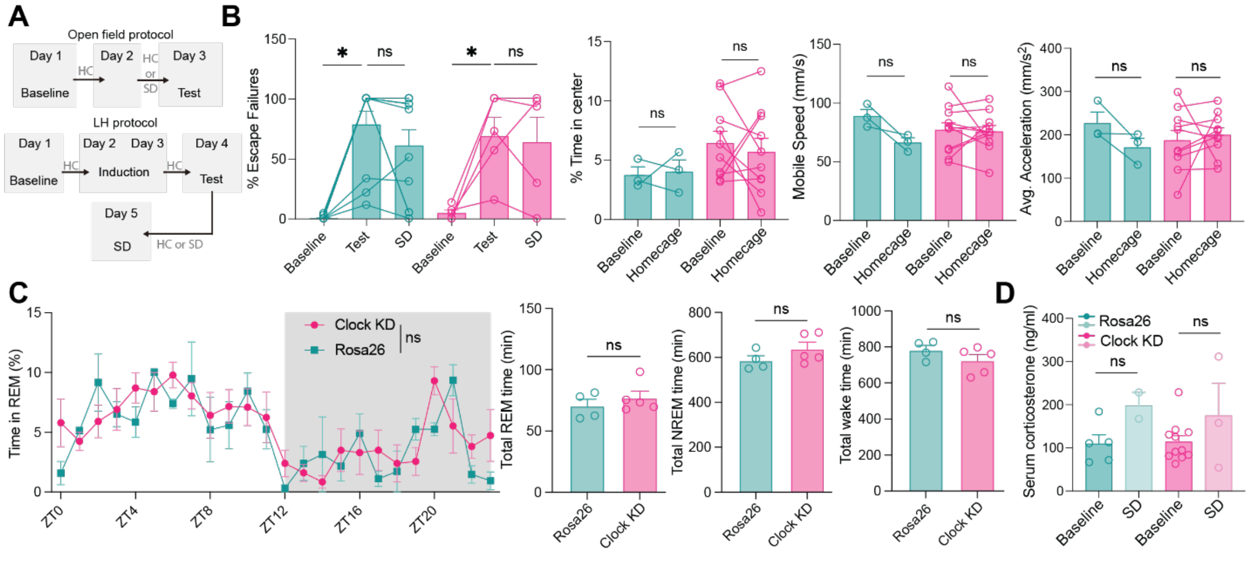

Supplementary Figure 3. (**A**) Timeline of open field and learned helplessness behavior protocols. (**B**) Anxiety and locomotion are unchanged in controls (n=3) compared to Clock KD (n=11) mice when animals are allowed to sleep in their homecage. (**C**) Clock disruption in VTA dopaminergic neurons does not alter serum corticosterone levels. Serum corticosterone concentrations (ng/mL) measured at ZT12 in Rosa26 and Clock KD mice at baseline (n=5, n=11 respectively) and immediately following 12 hours of sleep deprivation (SD; n=2, n=3 respectively). (**D**) Percent and total time spent in REM state is unchanged in mice injected with Clock KD (n=5) compared to control (n=4) virus during adulthood. Total time spent in NREM, and wake state are also not significantly different between Clock KD mice and controls. ns, not significant, *p<0.05.
